# Parathyroid hormone of ≥1.6 pmol/L at 6 months is associated with recovery in ‘long-term’ post-surgical hypoparathyroidism

**DOI:** 10.1530/ETJ-21-0130

**Published:** 2022-04-05

**Authors:** Muhammad Fahad Arshad, Amardass Dhami, Gillian Quarrell, Saba Prakash Balasubramanian

**Affiliations:** 1 Sheffield Teaching Hospitals NHS Foundation Trust, Sheffield, UK; 2 University of Sheffield, Sheffield, UK

**Keywords:** post-surgical hypoparathyroidism, recovery, parathyroid hormone, long-term

## Abstract

**Objective:**

Post-surgical hypoparathyroidism (PoSH) usually settles within few months after thyroid surgery, but several patients require long-term supplementation with calcium/activated vitamin D. When PoSH persists beyond 6 months, it is considered ‘chronic’ or ‘permanent’, however, late recovery has been reported. The aim of this study was to determine the frequency of late recovery and explore factors predicting late recovery of parathyroid function.

**Methods:**

Adult patients undergoing total/completion thyroidectomy between 2009 and 2018 were included in this retrospective cohort observational study. The records of patients with evidence of PoSH were reviewed to identify those with persisting PoSH at 6 months. Demographic, biochemical, surgical, pathological, and clinical follow-up data were collected and analysed.

**Results:**

Out of 911 patients undergoing thyroidectomy, 270 were identified with PoSH. Of these, 192 were started on supplements and 138 (71.9%) recovered within 6 months. Of the remaining 54 patients, 35 had ongoing PoSH with median (range) follow-up of 3.4 (0.5–11.1) years. Nineteen patients were weaned off supplements and achieved remission at median (range) follow-up of 1.3 (0.6–4.8) years. All of those who recovered had a PTH of ≥1.6 pmol/L at 6 months. There was no difference in age, gender, diagnosis, type, and extent of surgery between those who did and did not show late recovery.

**Conclusions:**

Recovery from PoSH is common beyond 6 months, raising the question whether a 6-month threshold to define ‘long-term’ PoSH is appropriate. The chances of recovery are high (~50%) in patients with PTH level ≥1.6 pmol/L at 6 months, where attempts at weaning may be focussed.

## Introduction

Hypoparathyroidism is defined as low calcium levels in presence of inappropriately normal or low parathyroid hormone (PTH) levels (1). It is commonly treated with calcium and activated vitamin D supplements, which makes it the only major endocrine disease, where hormone replacement is not standard practice. PTH replacement therapy may be useful if ‘the serum calcium level is poorly controlled presence of malabsorption, renal complications or poor quality of life’ but the quality of evidence supporting this is low (2). Hypoparathyroidism can result from several conditions, but the most common cause of hypoparathyroidism is neck surgery (3).

Post-surgical hypoparathyroidism or PoSH is the most common long-term complication after thyroidectomy. Hypocalcaemia appearing within first 24 h after surgery, reflecting parathyroid insufficiency, can occur in up to 30–60% of patients undergoing total thyroidectomy (1). In the majority of these patients (up to 90%), this is only transient, and recovery of parathyroid function (RPF) occurs, usually within the first few weeks to months (4). However, if hypoparathyroidism persists, this is termed ‘long term’, ‘chronic’, or ‘permanent’ (1, 5). The cut-off used for this distinction is usually 6 months as defined by the British and European guidelines (1, 6), although the American Association of Clinical Endocrinologists recommends extending this period to 12 months (5). The rate of developing long-term PoSH after thyroidectomy in the United Kingdom is 6% (6); however, the rates reported elsewhere are quite variable between 2 and 10% (7, 8). Several factors that predict the occurrence of long-term PoSH include low adjusted calcium after surgery, lack of identification of parathyroid glands at surgery, reoperation after bleeding, Graves’ disease, and large thyroid glands (9). Long-term hypoparathyroidism is associated with significant morbidity and mortality (10, 11). PoSH patients are at increased risk of renal complications (12, 13, 14), mental health problems (13, 15), and lower QOL (16). While the risk of ischemic heart disease, arrhythmias, and cataracts is increased in other hypoparathyroid patients, it is noteworthy that the patients with PoSH are relatively shielded from this (12). Some of the morbidity may be associated with the management of hypocalcaemia, particularly with the use of active vitamin D and calcium supplements. While these are needed to address the symptoms of hypocalcaemia, they increase calcium excretion and may occasionally cause hypercalcaemia.

Active vitamin D supplements may suppress PTH synthesis (17) and release and mask recovery of parathyroid function, unless weaning of treatment is actively considered. Patients with PoSH persisting beyond 6-12 months of surgery may not be considered for weaning of treatment as recovery after this time is traditionally considered unlikely.

However, recent studies have questioned this arbitrary cut-off of 6–12 months by showing recovery beyond this time (4, 18, 19, 20, 21). It is, however, unclear whether weaning should be pursued actively in patients with PoSH beyond 6–12 months and what factors (if any) can predict late recovery of parathyroid function (RPF).

The aim of this study was to determine the rate of late recovery of parathyroid function in a cohort of patients with long-term post-surgical hypoparathyroidism and to evaluate if there are any factors that predict this recovery.

## Methods

This was an observational retrospective cohort study of patients undergoing thyroidectomy (total or completion) at Sheffield Teaching Hospitals, UK between 2009 and 2018. The endocrine surgical unit at the hospital is a tertiary referral centre, and all operations are performed or supervised by a small team of experienced endocrine surgeons. Inclusion criterion was any adult patient over 18 years of age undergoing total or completion thyroidectomy for any condition. Patients undergoing surgery for recurrent disease and those undergoing concomitant parathyroid surgery were not included. The primary end point was RPF as reflected by complete weaning of calcium or activated vitamin D supplements.

All operations have been done using the traditional open incision. Central neck dissection was done alongside thyroidectomy in some patients with cancer and this was done in accordance with local protocols. Capsular dissection was employed as much as possible. Parathyroid glands were identified and preserved *in situ*where possible. Parathyroid auto-transplantation was performed selectively. No intraoperative techniques such as fluorescent imaging were used in this cohort.

A formal protocol was used to detect and manage post-thyroidectomy hypocalcaemia. This protocol has been modified over the course of the study, details of which have been published previously (22). In summary, patients had biochemical assessment on the day after surgery and treatment with calcium and/or vitamin D supplements were initiated selectively on the basis of low adjusted calcium, low PTH, and/or development of symptoms. Patients are then followed early (within 2 weeks of surgery), with further follow-ups planned at regular intervals depending on symptoms and biochemical assessments. In the first few weeks, the aim of treatment was to keep patients asymptomatic with adequate supplementation. Subsequent to this, weaning was attempted, when possible, but this was largely at the discretion of the treating clinician.

Data on demographics, indication and type of surgery, histological diagnosis, biochemical parameters, and medications were collected from paper and electronic records including primary care records. The indication for surgery was stratified as cancer or suspected cancer, hyperthyroidism (such as Graves’ disease), and nodular disease. The type of surgery was recorded as total thyroidectomy (TT) or completion thyroidectomy (CT), with or without central node dissection (CND), while the histological diagnosis was classified as benign or malignant. Patients’ hospital and primary care records were explored to confirm if and when they were weaned off calcium or activated vitamin D supplements, which was considered as RPF. The patients were followed up until their records confirmed RPF or December 31, 2020, whichever happened first. The biochemical data included adjusted calcium, PTH, and magnesium, and this was categorised into the following time intervals: day 1, between day 2 and 2 weeks, between 2 weeks and 3 months, between 3 and 6 months, and at ≥6 months. Date for serum creatinine were collected at surgery and at 6 months post-surgery. The biochemical data were validated by a second member of the team to improve the accuracy of the results. In order to identify all patients with post-surgical hypoparathyroidism, any patient who met at least one of the following three criteria on the day after surgery was included:

- PTH <1.6 pmol/L
- Adjusted calcium <2.1 mmol/L
- Initiation of treatment with calcium or activated vitamin D supplements

Serum calcium was measured using a Roche/Hitachi Cobas 8000 e702 automated clinical chemistry analyser (Roche Diagnostics GmbH). This method uses a 5-nitro-5’-methyl-BAPTA (NM-BAPTA) reagent. The inter-assay coefficient of variation as measured in the laboratory is 1.1–1.5% at 1.52 mmol/L and 0.6–1.1% at 3.07 mmol/L. Adjusted calcium was calculated by lab using equation:


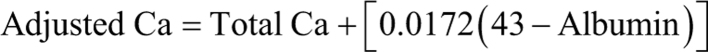


Intact PTH (second generation) was measured using an immunoassay method by the Roche Cobas 8000 e602 (Roche Diagnostics GmbH). The inter-assay coefficient of variation (CV) measured in the laboratory is 2.2–3.2% at 34 ng/L, 1.6–1.7% at 94 ng/L, and 1.4–1.8% at 839 ng/L. The normal ranges for serum adjusted calcium, plasma PTH, serum magnesium, and serum creatinine are 2.20–2.60 mmol/L, 1.6–6.9 pmol/L (15-65 ng/L), 0.7–1.0 mmol/L, and 44–80 µmol/L, respectively.

The data were collected on an Excel sheet and transferred to SPSS (version 26, IBM) for analysis. The continuous data were expressed as median (IQR) . Comparison of several variables between those who did and did not develop early and ongoing PoSH was done by using the chi-square (χ^2^) test (binary variables) and Mann–Whitney’s *U* test (continuous variables). As most of the patients had RPF within the first 3 years of follow-up, a separate comparison was also performed including only those with minimum 3 years of follow-up in the ongoing PoSH group. The time to RPF was analysed using Kaplan–Meier survival curve. The statistical significance for this study was set at *P* value of <0.05. The project’s ethical review was achieved by registrations with Sheffield Teaching Hospitals’ Clinical Effectiveness (reference number 10728) and Information Governance Units (reference number 3589).

## Results

**Figure 1:**
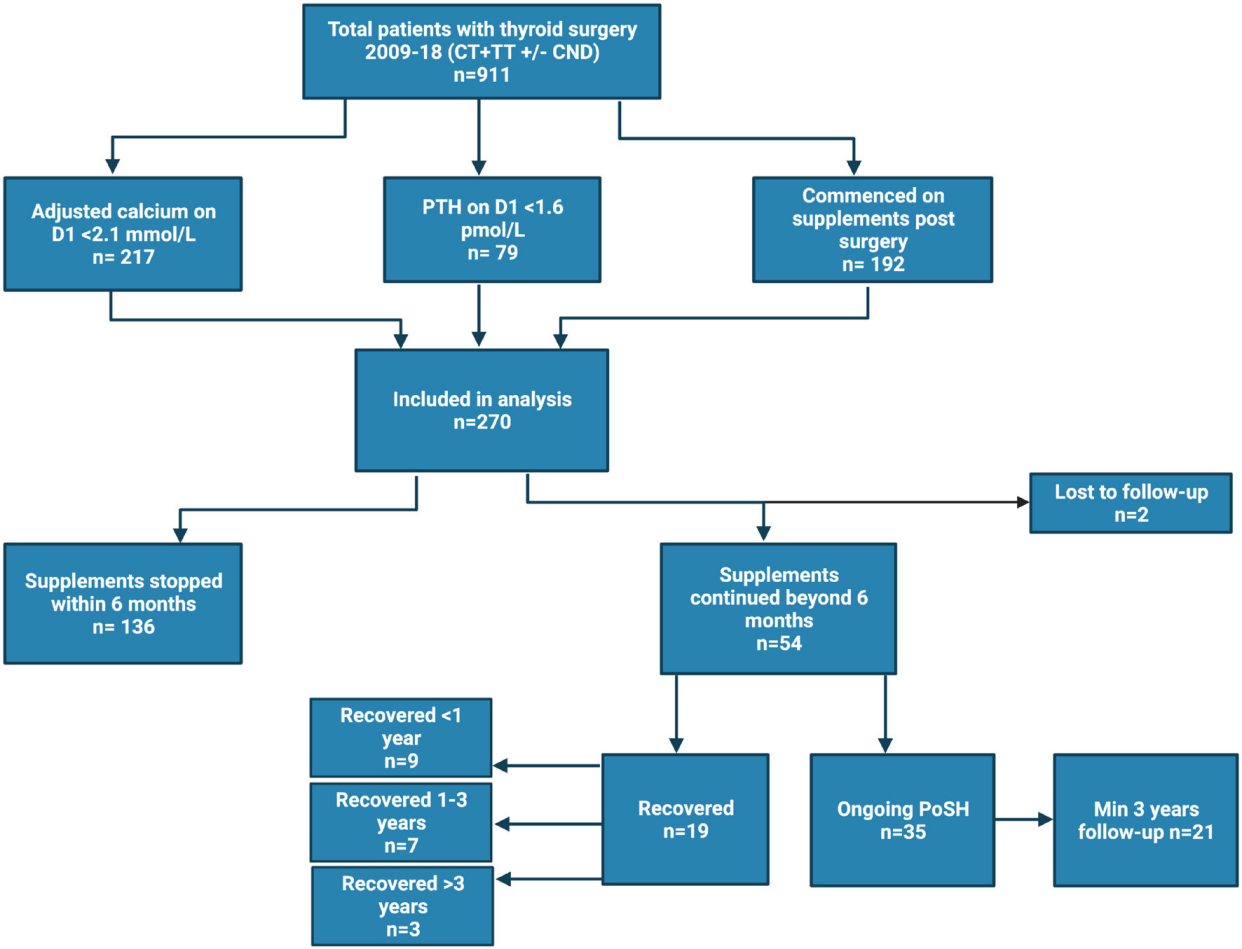
Study flow diagram. CND, central node dissection; CT, completion thyroidectomy; PoSH, post-surgical hypoparathyroidism; PTH, parathyroid hormone; TT, total thyroidectomy.

A total of 911 patients who underwent thyroidectomy between 2009 and 2018 were identified. Out of these, 270 patients (29.6%) met one or more of the defining criteria for PoSH. However, only 192 (21.1%) were commenced on supplements. Figure 1 shows the inclusion of patients in the study. The majority (79.6%) of patients were females with median (inter-quartile (IQR)) age of 45.2 years (IQR 32–57.6). The indications for the operations included hyperthyroidism (41.9%), cancer or suspected cancer (36.3%), and nodular disease (21.9%). Subsequently, histology confirmed cancer in nearly one-third of the cases. TT without CND was the most common surgery performed in this cohort (71.5%), followed by TT with CND (21.9%). The rate of CT with and without CND was 2.6% and 4.1%, respectively.

Of the total 192 patients commenced on supplements, the majority (70%; *n*  =136) had RPF within 6 months, while 54 patients were still on supplements at this cut-off point (5.9% of the overall cohort), and two patients were lost to follow-up. Table 1 compares the demographic, clinical, and biochemical parameters between these groups. While there were no differences in age, gender, pre-operative diagnosis, histology, and type of surgery between the two groups, PTH and adjusted calcium levels on the first postoperative day were higher in those who achieved RPF within 6 months compared to those with long-term PoSH.

**Table 1:** Demographic, clinical, and biochemical parameters of all patients developing PoSH and comparison of those who did or did not achieve remission at 6 months.

	Overall (*n* =270)	Recovery within 6 months (*n* = 136)	PoSH at 6 months (*n* = 54)	*P* value
Median age (IQR)	45.2 (32–57.6)	46.3 (33.9–57.6)	41.7 (28.9–59.9)	0.411
Gender, F/M	215/55	111/25	46/8	0.556
Pre-op diagnosis, % (*n*)				
Cancer/suspected cancer	36.3% (98)	38.2% (52)	42.6% (23)	0.739
Hyperthyroidism	41.9% (113)	40.4% (55)	40.7% (22)	
Thyroid nodule	21.9% (59)	21.3% (29)	16.7% (9)	
Pathology % (*n*)				
Benign	64.4% (174)	62.5% (85)	57.4% (31)	0.516
Malignant	35.6% (96)	37.5% (51)	42.6% (23)	
Type of surgery, % (*n*)				
CT	4.1% (11)	4.4% (6)	1.9% (1)	0.783
CT+CND	2.6% (7)	2.9% (4)	3.7% (2)	
TT	71.5% (193)	69.1% (94)	66.7% (36)	
TT+CND	21.9% (59)	23.5% (32)	27.8% (15)	
Adjusted calcium day 1 (mmol/L) (IQR) (*n*)	2.05 (1.98–2.09) (*n* =269)	2.06 (1.99–2.1) (*n* =136)	1.99 (1.90–2.07) (*n* =54)	**0.002**
PTH day 1 (pmol/L) (IQR) (*n*)	2.0 (1.0–3.3) (*n* = 190)	2.3 (1.4–3.5) (*n* = 113)	0.8 (0.5–1.2) (*n* = 39)	<0.001

CND, central node dissection; CT, completion thyroidectomy; F/M, female/male; IQR, inter-quartile range; PoSH, post-surgical hypoparathyroidism; PTH, parathyroid hormone; TT, total thyroidectomy.

Of the 54 patients with long-term PoSH, 19 patients were completely weaned off their supplements and were considered to have recovered their parathyroid function (RPF). Most of the patients had RPF within the first 3 years (nine between 6 and 12 months and seven between 1 and 3 years), while 35 patients were still on supplements until the end of the study period and were therefore deemed to have persistent PoSH. Of these, 21 had PoSH beyond 3 years of follow-up while the remaining (*n*  = 14) were within 3 years of follow-up. Table 2 compares the demographic, clinical, and biochemical parameters between groups with and without late (beyond 6 months) recovery of parathyroid function. There was no difference in demographics, surgical, and other clinical parameters between the two groups. PTH and adjusted calcium levels on the day after surgery and at subsequent times until 6 months were also similar between the two groups. But the adjusted calcium and especially PTH at or beyond 6 months was significantly higher in patients with recovery of function. When the patients were categorised according to whether they had PTH of ≥1.6 pmol/L (lower limit of normal reference range) at this time period, all of the those who recovered had PTH above this cut-off compared to 16/25 (64%) of the patients in the ongoing PoSH group (*P*  = 0.008) (sensitivity 100%; specificity 40%). Further ROC curve analysis (results not shown here) confirmed that the 100% sensitivity can also be maintained up to a PTH threshold of 1.8 pmol/L with corresponding specificity of 52%. The median follow-up time for patients with ongoing PoSH was 3.4 years.

**Table 2:** Comparison of demographic, clinical, and biochemical parameters between those who did or did not achieve remission beyond 6 months.

	Late recovery (beyond 6 months) (*n* = 19)	Persistent PoSH without evidence of late recovery (*n* = 35)	*P* value
Median age (IQR)	39.6 (27.2–51.9)	47.3 (29.5–68.9)	0.113
Gender F/M	18/1	28/7	0.145
Pre-op diagnosis % (n)			
Cancer/suspected cancer	31.6% (6)	48.6% (17)	0.393
Hyperthyroidism	52.6% (10)	34.3 % (12)	
Thyroid nodule	15.8% (3)	17.1% (6)	
Pathology, % (*n*)			
Benign	68.4% (13)	51.4% (18)	0.228
Malignant	31.6% (6)	48.6% (17)	
Type of surgery, % (*n*)			
CT	0%	2.9% (1)	0.600
CT+CND	0%	5.7% (2)	
TT	73.7% (14)	62.9% (22)	
TT+CND	26.3% (5)	28.6% (10)	
Adjusted calcium day 1 (mmol/L) (IQR) (*n*)	1.95 (1.84-2.05) (*n* = 19)	2.01 (1.93-2.08) (*n* = 35)	0.198
Adjusted calcium day 2 to 2 weeks (mmol/L) (IQR) (*n*)	1.93 (1.85–2.02) (*n* = 19)	1.99 (1.90–2.15) (*n* =34)	0.185
Adjusted calcium 2 weeks to 3 months (mmol/L) (IQR) (*n*)	2.23 (2.13–2.31) (*n* =18)	2.18 (2.0– 2.27) (*n* =34)	0.240
Adjusted calcium 3–6 months (mmol/L) (IQR) (*n*)	2.20 (2.11–2.22) (*n* = 16)	2.19 (2.02–2.26) (*n* = 31)	0.928
Adjusted calcium >6 months (mmol/L) (IQR) (*n*)	2.17 (2.07–2.26) (*n* = 16)	2.09 (1.97–2.18) (*n* = 31)	**0.048**
PTH day 1 (pmol/L) (IQR) (*n*)	0.9 (0.7–1.4) (*n* = 13)	0.8 (0.5–1.0) (*n* = 26)	0.435
PTH day 2 to 2 weeks (pmol/L) (IQR) (n)	0.8 (0.5–1.6) (*n* = 16)	0.9 (0.6–1.2) (*n* =27)	0.554
PTH 2 weeks to 3 months (pmol/L) (IQR) (*n*)	2.2 (1.5–3.6) (*n* = 15)	1.4 (0.8–2.2) (*n* = 28)	0.072
PTH 3–6 months (pmol/L) (IQR) (*n*)	2.3 (1.8–3.8) (*n* =14)	1.8 (0.8–2.5) (*n* = 23)	0.071
PTH ≥6 months (pmol/L) (IQR) (n)	2.6 (2.3–3.0) (*n* = 15)	1.8 (1.2–3.0) (*n* = 25)	**0.016**
PTH ≥1.6 pmol/L at >6 months	15/15	16/25	**0.008**
Magnesium day 1 (mmol/L) (IQR) (*n*)	0.68 (0.57–0.69) (*n* = 6)	0.71 (0.67–0.77) (*n* =13)	0.127
Magnesium day 2 – 2 weeks (mmol/L) (IQR) (*n*)	0.66 (0.59–0.75) (*n* = 6)	0.74 (0.68–0.79) (*n* = 19)	0.198
Magnesium 2 weeks to 3 months (mmol/L) (IQR) (*n*)	0.77 (0.71–0.81) (*n* = 6)	0.8 (0.76–0.84) (*n* = 19)	0.246
Magnesium 3–6 months (mmol/L) (IQR) (*n*)	0.81 (0.75–0.86) (*n* = 6)	0.8 (0.76–0.87) (*n* = 17)	1.0
Magnesium >6 months (mmol/L) (IQR) (n)	0.82 (0.76–0.87) (*n* = 11)	0.8 (0.76–0.85) (*n* = 29)	0.720
Creatinine at surgery (µmol/L) (IQR) (*n*)	63 (52–78) (*n* = 19)	67 (55–80) (*n* = 34)	0.388
Creatinine >6 months (µmol/L) (IQR) (*n*)	70 (62–78) (*n* = 19)	72 (64–85) (*n* = 34)	0.220

CND, central node dissection; CT, completion thyroidectomy; F/M, female/male; IQR, inter-quartile range; PoSH, post-surgical hypoparathyroidism; PTH, parathyroid hormone; TT, total thyroidectomy.

While most of the patients with recovery of parathyroid function had this occur within the first 3 years, there were several patients in the ongoing PoSH group who had a follow-up of less than 3 years. After excluding those with <3 years of follow-up (*n*  = 21), the analysis was repeated. The PTH level at or after 6 months, however, remains significant (Supplementary Table 1, see section on supplementary materials given at the end of this article).

**Figure 2:**
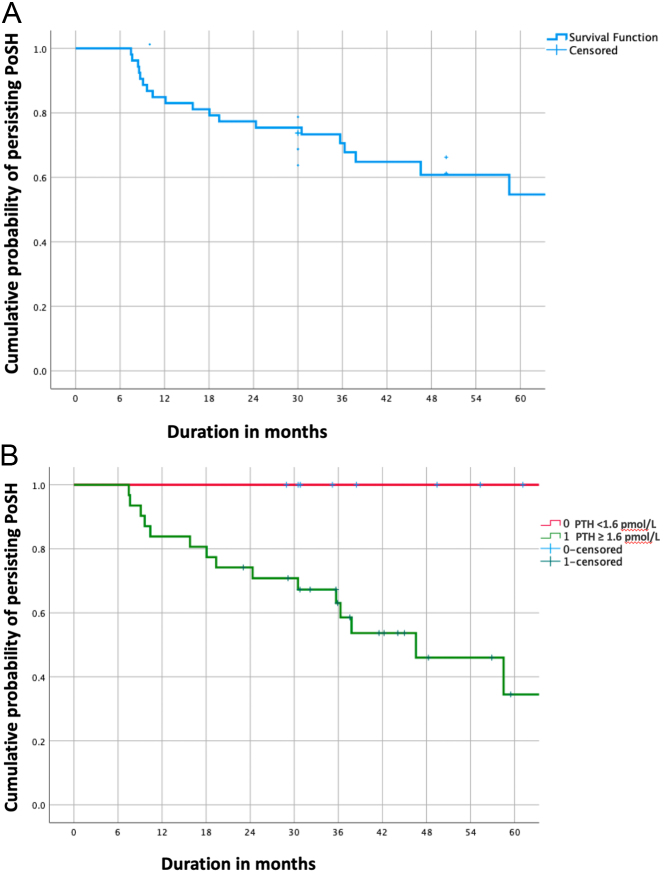
(A) Kaplan–Meier curve showing recovery of parathyroid function beyond 6 months in all patients with PoSH at 6 months and (B) Kaplan–Meier curve showing recovery of parathyroid function beyond 6 months stratified by PTH of <1.6 pmol/L or ≥1.6 pmol/L.

Figure 2 shows a Kaplan–Meier curve showing RPF over time in patients with long-term PoSH. As seen in the figure, the longest recovery in this cohort occurred at around 5 years after surgery.

## Discussion

The results of this study confirm previously observed findings that recovery from PoSH is a dynamic process and can occur even after 6 months, and in some cases, several years after surgery (4, 18, 19, 20, 21). While time thresholds are helpful in formulating definitions for long-term outcomes (such as PoSH) to enable comparisons of the results across centres and studies, these results question whether the cut-off time for long-term PoSH should be extended. In addition, guidance on long-term PoSH should emphasize that recovery can still occur after the cut-off time and the terms ‘chronic’ and ‘permanent’ should be avoided when referring to long-term PoSH.

The study also illustrates a novel finding that if the PTH level at or after 6 months is within normal range, then there is almost 50% likelihood of recovery in the ensuing 3 years. This makes PTH a useful biomarker in clinical practice as these patients can be actively tried to wean off the supplements. This has been shown earlier in a small study from Korea in 2015 (21). In this study, 5 out of 22 patients who made RPF after 12 months had their PTH within normal range. For patients with low PTH at 6 months, it is not clear if recovery is still possible in the long term.

Late recovery of parathyroid function has been described previously. Villarroya-Marquina *et al.* reported in 2018 that of 69 patients with PoSH at 6 months, 21 patients recovered within 6–12 months and another 12 were weaned off the supplements after 12 months, compared to 36 who had ongoing PoSH (18). In another study in 2020 including 71 adults and 23 children with 6 months PoSH, recovery was reported in 45 patients after 6 months, while 49 patients still remained on supplements (4). A recent Chinese study (2021) reported on a large cohort of patients with papillary cancer (*n*  = 968); 42 of whom had PoSH at 6 months. They demonstrated late recovery of function in 19 patients (*n*  = 11 between 6–12 months and *n*  =8 after 1 year), compared to 23 patients who remained dependent on supplements (23). In patients where recovery was late, the longest recovery time has not been clearly reported in these studies. The mechanisms underlying late recovery are subject to speculation. This could be related to lack of implementation of aggressive weaning strategies or the volume of residual, potentially viable parathyroid tissue following surgery.

An adjusted calcium at 1 month of >2.25 mmol/L has been reported as a predictor of late recovery in two studies (4, 18). In contrast, the study from China suggested a much lower cut-off of >2.07 mmol/L to predict the same outcome (23). This study, in contrast, shows that there was no difference in calcium levels between those with late recovery and ongoing PoSH at any point within first 6 months. The adjusted calcium between 2 weeks and 3 months which will roughly correspond to the same time period was 2.23 mmol/L (2.13–2.31) in those who recovered versus 2.18 mmol/L (2.0–2.27) in those who continued on supplements (*P*  = 0.240). Adjusted calcium is not likely to be a reliable predictor of late recovery as the extent of treatment is likely to influence adjusted calcium levels and there is controversy over whether patients should be under treated (parathyroid stimulation) or adequately treated (parathyroid splinting) to facilitate recovery. None of these studies (4, 18, 23) have reported on the predictive value of biochemical markers (including PTH) at the defined threshold for long-term PoSH (around 6–12 months) to provide a comparison for our findings.

The other important predictive factor for RPF described in literature is the parathyroid glands remaining *in situ* (PGRIS) score (4, 18). We did not measure this score in our study as this score has several limitations. For example, the score does not take into account supernumerary parathyroid glands (24, 25), vascularity or function of otherwise normal-looking glands. Besides, the accuracy of the score is also dependent on expertise of the pathologist (26).

The high rate of recovery of parathyroid function in our study may reflect aggressive attempts to wean patients off supplements. This is likely to be of long-term benefit to patient as it avoids hypercalcaemia, hypercalciuria, and other morbidity related to treatment of PoSH. Maintaining calcium levels below reference range does not affect bone health either, as PoSH is associated with low bone turnover and increased bone mineral density (27) and protection against fractures (28), however, a recent observational study has shown increased risk of vertebral fractures in postmenopausal women with PoSH (29).

This study is a single-centre observational study and therefore has limitations inherent with this type of study design such as missing data, risk of ascertainment bias and the potential lack of generalizability. It is also worth mentioning here that the rate of recovery of parathyroid function could vary among various clinicians and plausibly higher when more aggressive strategies to wean off supplements are employed. The term ‘recovery of parathyroid function’ may be controversial but refers to the ability to wean patients off calcium and/or active vitamin D supplements without precipitating symptoms of hypoparathyroidism or a significant fall in calcium and/or PTH levels (4, 18, 23). While the study included all patients at risk of PoSH after thyroid surgery over a 10-year period, the number of patients with 6 months PoSH is still relatively small. Lastly, precise data on treatment regimens and the intention to either splint (adequately treat hypocalcaemia) or stimulate (under treat hypocalcaemia) was not available.

To conclude, late parathyroid recovery beyond 6–12 months is not rare. The term ‘chronic’ or ‘permanent’ post-surgical hypoparathyroidism should be avoided and the potential for late recovery should be considered in revised guidelines. PTH is a useful biomarker in predicting long-term recovery of parathyroid function.

## Supplementary materials

Supplementary table 1: Comparison of demographic, clinical, and biochemical parameters between those who did or did not achieve remission beyond six months (minimum three years follow up)

## Declaration of interest

All other authors declare that there were no conflicts of interest that could be perceived as prejudicing the impartiality of the research reported. Saba Balasubramanian is a member of the editorial board of *European Thyroid Journal*. Saba Balasubramanian was not involved in the review or editorial process for this paper, on which they are listed as an author.

## Funding

This work did not receive any specific grant from any funding agency in the public, commercial, or not-for-profit sector.

## Data availability

The data supporting these findings is available within the article. Raw data that support the findings of this study are available from the corresponding author upon reasonable request.

## Author contribution statement

S P B conceived this study. S P B, M F A, and A D developed the protocol and study data collection tools. Data collection was performed by M F A, A D, and G Q. Analysis was performed by M F A and S P B. The first draft of the manuscript was jointly prepared by M F A with critical input on multiple versions of the manuscript from all authors. All authors approved the final submitted version. M F A and S P B are the guarantors of this work, and as such had full access to all study data and thus take responsibility for data integrity and accuracy of data analysis.
