# Supplementary material for: Parathyroid hormone of ≥1.6 pmol/L at 6 months is associated with recovery in ‘long-term’ post-surgical hypoparathyroidism
Source: Eur Thyroid J. 2022 Apr 5;11(3):e210130. doi: 10.1530/ETJ-21-0130 (PMC9175593; doi:10.1530/ETJ-21-0130)
Supplement: Supplementary table 1: Comparison of demographic, clinical, and biochemical parameters between those who did or did not achieve remission beyond six months (minimum three years follow up) [file supplementary_table_1.pdf]

Supplementary table 1: Comparison of demographic, clinical, and biochemical parameters between those who did or did not achieve remission beyond six months (minimum three years follow up)

	Late recovery (beyond 6 months) (n=19)	Persistent PoSH without evidence of late recovery with 3 years follow up (n=21)	p value
Median Age [IQR]	39.6 [27.2-51.9]	47.5 [36.6-62.1]	0.065
Gender F/M	18/1	17/4	0.188
Pre-op diagnosis % (n)<br>Cancer/ suspected cancer<br>Hyperthyroidism<br>Thyroid nodule	 31.6% (6)<br>52.6% (10)<br>15.8% (3)	 42.9% (9)<br>38.2% (8)<br>19% (4)	 0.648
Pathology % (n)<br>Benign<br>Malignant	 68.4% (13)<br>31.6% (6)	 57.1% (12)<br>42.9% (9)	 0.462
Type of surgery % (n)<br>CT<br>CT+CND<br>TT<br>TT+CND	 0%<br>0%<br>73.7% (14)<br>26.3% (5)	 0% (0)<br>4.8% (1)<br>61.9% (13)<br>33.3% (7)	 0.529
Adjusted calcium day 1 (mmol/L) [IQR] (n)	1.95 [1.84-2.05] (n=19)	2.01 [1.92-2.1] (n=21)	0.226
Adjusted calcium day 2 – 2 weeks (mmol/L) [IQR] (n)	1.93 [1.85-2.02] (n=19)	2.01 [1.88-2.16] (n=20)	0.214
Adjusted calcium 2 weeks- 3 months (mmol/L) [IQR] (n)	2.23 [2.13-2.31] (n=18)	2.20 [2.07-2.28] (n=20)	0.613
Adjusted calcium 3-6 months (mmol/L) [IQR] (n)	2.20 [2.11-2.22] (n=16)	2.19 [2.1-2.25] (n=18)	0.905
Adjusted calcium >6 months (mmol/L) [IQR] (n)	2.17 [2.07-2.26] (n=16)	2.14 [2.05-2.22] (n=18)	0.297
PTH day 1 (pmol/L) [IQR] (n)	0.9 [0.7-1.4] (n=13)	0.8 [0.5-1.4] (n=16)	0.779

**PTH day 2 – 2 weeks (pmol/L) [IQR] (n)**	0.8 [0.5-1.6] (n=16)	1.0 [0.6-1.2] (n=16)	0.515
**PTH 2 weeks- 3 months (pmol/L) [IQR] (n)**	2.2 [1.5-3.6] (n=15)	1.6 [0.9-2.2] (n=16)	0.163
**PTH 3-6 months (pmol/L) [IQR] (n)**	2.3 [1.8-3.8] (n=14)	2.0 [0.6-2.6] (n=12)	0.106
**PTH ≥6 months (pmol/L) [IQR] (n)**	2.6 [2.3-3.0] (n=15)	1.9 [1.2-2.5] (n=15)	**0.023**
**PTH ≥1.6 pmol/L at >6 months**	15/15	10/15	**0.014**

Abbreviations: PoSH= Post- surgical hypoparathyroidism; IQR= Inter-Quartile range; F/M= Female/Male; CT= Completion thyroidectomy; CND= Central node dissection; TT= Total thyroidectomy; PTH= Parathyroid hormone
